# Effect of growth hormone on bone fragility in patients with Prader-Willi syndrome scoliosis

**DOI:** 10.1186/1748-7161-10-S1-O10

**Published:** 2015-01-19

**Authors:** Yutaka Nakamura, Nobuyuki Murakami, Takahiro Iida, Satoshi Asano, Satoru Ozeki, Kanta Tajima, Masayoshi Kanai, Toshiro Nagai

**Affiliations:** 1 Spine Centre, Higashi Saitama General Hospital, Japan; 2 Department of Paediatrics, Dokkyo Medical University Koshigaya Hospital, Japan; 3 Department of Orthopaedic Surgery, Dokkyo Medical University Koshigaya Hospital, Japan

## Objective

Patients with Prader-Willi syndrome (PWS) is known to have bone fragility. Osteoporosis may be associated with bone fractures and poor surgical outcome for scoliosis. Current growth hormone (GH) treatment offers a viable and safe means to reduce these risks by improving bone density. The purposes of this study were to investigate bone mineral density (BMD) with PWS patients and to verify efficacy of growth hormone (GH) administration for osteoporosis.

## Methods

We investigated 148 patients (91 male, 57 females) with PWS who examined BMD and followed for at least 2 years at our out-patient clinic. Measurement was taken at the lumbar spine (L2-4). The mean age was 9.35 (2-47) years at initial BMD. Scoliosis was found in 64 PWS patients (GH plus, 45; minus, 19). Mean cobb angle was 30.6 (range 12-88) degrees. There were 84 non-scoliosis patients (GH plus; 51, GH minus; 33). Patients were treated with GH (0.245 mg/kg/week) over a period of 3 to 16 years (mean, 5 years, 11 months). We also evaluated the effect of GH treatment on BMD in 108 patients who underwent BMD testing more than twice (GH plus, 89 patients; GH minus, 19 patients).

We focused on the following:

1) Measuring the lumbar BMD before GH treatment and evaluated the prevalence of bone fragility (osteoporosis and osteopenia) in all patients.

2) Retrospectively evaluating the association between scoliosis and lumbar BMD.

3) Comparing the BMD between at initial and at final follow-up stage to determine the effect of GH treatment for the increase of BMD.

## Results

1). Total lumbar BMD (L2-4) was 0.567 g/cm (Z score -2.12). Fifty (33.8%) patients had osteoporosis (Z score <-2.5) and 41 (27.7%) patients had osteopenia (-1.5< Z score<-2.5). Total 61.5% of patients showed bone fragility (osteoporosis and osteopenia).

2). The scoliosis group was 0.598 g/cm (Z score -2.08) and non-scoliosis group was 0.548 g/cm (Z score -2.27). There were no significant differences between the two Groups (P=0.075).

3). GH treatment revealed a significant increase of L2-4 BMD (GH plus; 7.95%VS GH minus: 2.57%, P<0.01).

## Conclusions

1) 61.5 % of patients with PWS had bone fragility. 2) The frequency of scoliosis was not correlated with BMD.3) GH administration significantly improved BMD. GH therapy may be a useful treatment for reducing the frequency of bone fractures and surgical risk in PWS patients associated with osteoporosis.

